# Effects of multiple photon scattering on maximum likelihood positioning in PET

**DOI:** 10.1186/2197-7364-1-S1-A18

**Published:** 2014-07-29

**Authors:** Nicolas Groß-Weege, David Schug, Volkmar Schulz

**Affiliations:** Department of Physics of Molecular Imaging Systems, Institute for Experimental Molecular Imaging, RWTH Aachen University, Aachen, Germany; Philips Research Laboratories, Aachen, Molecular Imaging Systems, Kragujevac, Germany

Conventional positioning algorithms in PET assume a photon interaction and complete energy deposition in one detector element (single-hit). However, there is a high probability that a photon interacts within a crystal element via Compton scattering but is absorbed in another element (multiple-hit). Thus scintillation light is generated in multiple crystals and the resulting light distribution is spread over more detector elements. This leads, among others, to a degradation of the spatial resolution, especially in pixelated scintillators with a small pitch, unless more sophisticated positioning algorithms are exploited.

In this work, we use a maximum-likelihood method (MLM) to identify the 2D hit position with a single-hit probability model. We want to study the characteristics of multiple-hit events, to implement a rejection filter using a likelihood threshold, which will consequently be beneficial for the intrinsic spatial resolution.

For the studies we use a sensor tile with 8×8 digital SiPMs. A 30×30 LYSO crystal array (crystal volume 1×1×12mm³) is mounted on top of the tile using an intermediate light guide (2mm).

This setup was used to measure the single-hit probability density for the MLM using a constrained center-of-gravity positioning. To study the MLM, we simulate the different event classes using the above described setup with an additional 511keV point source.

The response of the SiPMs to a simulated event is modeled in two steps. First the deposited energy is determined for each hit crystal with intrinsic energy resolution and converted into photon count. Second this photon count is spread over the SiPMs with the related mean of the measured probability density. For multiple-hit events, the calculated photon count of all hit crystals for each SiPM is summed up.

We are able to demonstrate, that a likelihood threshold can substantially improve the detector intrinsic spatial resolution of blurring caused by multiple-hit events. Results and details about this study will be presented at the conference.

